# Role of Non-Coding RNAs in the Transgenerational Epigenetic Transmission of the Effects of Reprotoxicants

**DOI:** 10.3390/ijms17040452

**Published:** 2016-03-25

**Authors:** Eduardo Larriba, Jesús del Mazo

**Affiliations:** Department of Cellular and Molecular Biology, Centro de Investigaciones Biológicas (CSIC), Ramiro de Maeztu 9, Madrid 28040, Spain; larriba.ed@gmail.com

**Keywords:** reproduction, reprotoxicology, reprotoxicants, non-coding RNAs, epigenetics, transgenerational transmission, endocrine disruptors, microRNAs, piRNAs, lncRNAs

## Abstract

Non-coding RNAs (ncRNAs) are regulatory elements of gene expression and chromatin structure. Both long and small ncRNAs can also act as inductors and targets of epigenetic programs. Epigenetic patterns can be transmitted from one cell to the daughter cell, but, importantly, also through generations. Diversity of ncRNAs is emerging with new and surprising roles. Functional interactions among ncRNAs and between specific ncRNAs and structural elements of the chromatin are drawing a complex landscape. In this scenario, epigenetic changes induced by environmental stressors, including reprotoxicants, can explain some transgenerationally-transmitted phenotypes in non-Mendelian ways. In this review, we analyze mechanisms of action of reprotoxicants upon different types of ncRNAs and epigenetic modifications causing transgenerationally transmitted characters through germ cells but affecting germ cells and reproductive systems. A functional model of epigenetic mechanisms of transgenerational transmission ncRNAs-mediated is also proposed.

## 1. Introduction

The notion that most of the genome in the Eukaryota was “junk DNA” is already part of Biology history. Projects such as ENCODE [1] and the FANTOM Consortium [2], using powerful methodological approaches as Next Generation Sequencing (NGS) followed by bioinformatic tools, are revealing that most of DNA is transcribing in RNAs that does not code proteins. These non-coding RNAs (ncRNAs) have crucial regulatory functions in cells, development and differentiation [3], including reproductive systems [4]. Consequently, alterations of normal expression or mutation of such regulatory elements could end in pathologies [5,6].

The world of ncRNAs is expanding in diversity of types, biogenesis, mechanisms of action and functions. The complexity of all functional variants is enhanced by the diversity of cell types and developmental periods but also mediated by environmental mechanisms. At present, both small and long non-coding RNAs are considered key elements in the fine regulatory mechanisms of gene expression and function. Different functional types of small non-coding RNAs (sncRNAs) (about 18–35 nucleotides) have been reported in animals. The most representatives are microRNAs (miRNAs), Piwi-interacting RNAs (piRNAs) and endogenous-small interfering RNA (endo-siRNAs). miRNAs are best characterized in their biogenesis and functions [7]. However, seminal works characterized the roles of some particular long non-coding RNAs (lncRNAs) (more than 200 nucleotides). This is the case of the *Xist* gene that participates, by epigenetic mechanisms, in the inactivation of one X chromosome in females or the X chromosome in spermatogenesis [8,9,10,11], or other lncRNAs involved in genomic imprinting [12].

Changes in environmental conditions have been considered as a cause of changes in the phenotype of affected individuals and populations. The effects can be triggered by both genetic and epigenetic mechanisms, considering epigenetics as the heritable changes in genome function, affecting gene expression and genome activity, without changes in DNA sequences [13]. Environmental stressors, inducing adverse effects, can act from diverse and complex mechanisms of action. The changes in the genome expression modulated by environmental conditions are mediated by epigenetic mechanisms able to induce alterations in gene expression with phenotypic effects in individuals exposed, and with the possibility of transmission to successive generations [14]. Reprotoxicants are considered those toxicants that in someway affect reproductive systems of the organisms.

Toxicological adverse effects of environmental pollution in reproductive systems of human and animal organisms are generating wide social concern. This worry was mainly based on the epidemiological data of reproductive disorders specially detected in males, both in spermatogenesis, gonadal development and testicular cancer under the called testicular dysgenesis syndrome (TDS) [15,16]. The different nature of toxicants causing alterations of the reproductive systems can adversely impact development in different ways. However, a common endpoint in the mechanisms causing altered phenotypes is deregulation of gene expression of the genes involved in reproductive biological processes. A cornerstone in the assessment of potential adverse effects of a huge amount of different environmental reprotoxicants is to understand its molecular mechanisms of action. The most vulnerable developmental windows could be the embryonic period that along with the potential transgenerational transmission of deleterious phenotype, is being detected as the effect of some toxicants, are increasing the health and social concerns.

Hundreds of chemical or physical elements have been reported as potential toxicants as a consequence of environmental exposure, inducing epigenetic modifications [17]. In some of these compounds, clinical and epidemiological effects have been identified and their mechanisms of action as reprotoxicants scientifically tested. Probably the most widely studied are the compounds considered as endocrine disruptors (EDs) [18,19,20,21,22]. Here, we will review different mechanisms of epigenetic transmission environmentally induced, focusing on the role of different non-coding RNAs in the alteration of germ cells differentiation and reproductive systems development, caused by the exposure of environmental pollutants, which by epigenetic mechanisms could be transmitted to successive generations.

## 2. Mechanisms of Epigenetic Transmission of Adverse Effects

The health risk of potential environmental toxicants and their control is included in most prevention programs. However, the fear of transmission to successive generations of deleterious characters is a also social phenomenon. We should differentiate intergenerational transmission that only affect the first offspring generation of the exposed individual from transgenerational transmission when successive offspring generations inherit the phenotypic traits [23]. It is logical to understand that if a toxicant is able to induce mutations on DNA, of those affected, the germ line can be Mendelian transmitted to the next generations, including DNA sequences of both coding and non-coding genes. This category includes reprotoxicants. However, such mutations affecting reproduction phenotypes are, by the nature of the affected system, under high negative selection. Epimutations are mutations that affect phenotypes without modifications in DNA sequences. Transgenerational inheritance of phenotypes induced by environmental stress is a concept closely associated with epigenetics [24].

Due to its own nature, the epigenetic modifications can be considered as mechanisms of response to environmental changes of living conditions for the organisms. Hence, epigenetic changes in germ cells show the highest rate of modification by environmental elements but also the highest rate of reversibility. Changes in the patterns of gene expression in developmental gonads after parental exposure have been considered as potential changes in the epigenetic marks [25]. Three major mechanisms are involved in epigenetic changes: DNA methylation, chromatin remodeling by histone modifications, and, more recently, epigenetic gene regulation by ncRNAs.

### 2.1. DNA Methylation

DNA methylation is one of the most analyzed epigenetic marks. DNA methylation is a covalent binding of methyl chemical groups to cytosine residues. The DNA is methylated by methyltransferases in cytosine of, mainly but not exclusively, the CpG dinucleotide sequences concentrated in CpG islands of many gene promoter regions. DNA methylated is associated with transcription repression. However, other forms or variants of chemical epigenetic modifications of the cytosine residues are being considered in establishment of regulatory epigenetic patterns of gene expression such as hydroxymethylation, carboxylation and formylation [26,27,28]. However, the role of these different epigenetic marks on cytosine are not fully established, neither how a defined mixture of different marks, developmental or environmentally induced, can be transgenerational inheritable.

In mammals, the maternal or paternal genome patterns of DNA methylation in germ cells have to be reprogrammed after fertilization to differentiating cells and tissues. During the development and germ cell differentiation, reprogramming of DNA methylation occurs in diverse waves of methylation and demethylation. In mammals, primordial germ cell (PGCs), the founder cells of gametes, suffer a process of demethylation with a minimum level of methylated DNA in the stage before entry into meiosis in female and mitotic arrest in males [29,30]. Later, in the differentiation of both female and male germ cells, *de novo* remethylation occurs [31,32].

However, the pattern of DNA methylation can be maintained without reprogramming in some regions of the chromatin. Such established marks of DNA methylated or demethylated can be the origin of epigenetic transmission, the consequences in gene expression and the associated phenotypes. The most conspicuous examples of DNA escaping reprogramming of methylation patterns of erasure and resetting are the imprinted genes—that is, the alleles that in somatic cells maintain the maternally or paternally inherited patterns [33]. Many other characterized genome regions are not following the static patterns of methylation reprogramming, neither the paternal nor maternal genome display the same events in methylation, especially during gametogenesis and early embryogenesis [34,35]. This landscape indicates that methylation and associated mechanisms are dynamic processes able to be adapted to specific requirements of development and potentially to the changes in the environment, especially in early embryonic stages and germ cell differentiation, including stress induced by reprotoxicants.

Some DNA methylation changes in reproductive system have been attributed to the exposure to EDs. One of the most widely EDs studied was diethylstilbestrol (DES), tragically famous by the correlation between mothers exposure, to prevent miscarriage and other pregnancy complications, and the incidence of vaginal adenocarcinoma in their young daughters [36,37]. More recently, epigenetic changes in the expression of DNA methyltransferases (DNMTs) catalyzing methylation of genomic DNA have been reported in animal models, as they are involved in DES effects during perinatal exposure [38].

Changes in the DNA methylation pattern have been directly related to transgenerational effect of reprotoxicants. Environmental exposure to different compounds, mainly EDs, have been attributed as the cause of modification in the developmental patterns of DNA methylation, mainly in the CpG islands, and, consequently, in alterations in the pattern of gene expression [39], including testicular cancer [40]. Most of the methylation changes and transgenerational transmission of epimutations induced by reprotoxicants [41] have been associated with the male germ line [42]. However, differential DNA methylation pattern in males could be transgenerationally inherited through the female germline, as suggested in the assessment of the effects of a pesticide used as a substitute of DDT: methoxychlor [43].

### 2.2. Chromatin Histone Code

In mammals, chromatin is integrated by nucleosomes, which consists of DNA wrapped twice around a histone octamer, containing each two copies each of four highly conserved histones: H2A, H2B, H3 and H4. Histone tails on the nucleosome are subject to post-translational modifications processes of selected amino acids including methylation, acetylation, phosphorylation, ubiquitination, biotinylation, sumoylation and ADP-ribosylation. These modifications are associated with changes in both activation and repression of transcription. The “histone code” regulates the structure and function of chromatin. These enzyme-mediated post-translational modifications generate epigenetic codes that specify different patterns of gene expression generating active and repressive marks. The deregulation mediated by environmental factors of histone-modifying enzymes such as histone acetyltransferases, histone deacetylases, histone methyltransferases and histone demethylases could alter the functional structure of chromatin and hence normal physiology. Environmental contaminants were demonstrated to enhance the histone acetyltransferase activity [44] or to inhibit histone deacetylase activity [45]. The code established during development and cell differentiation is transmitted through cell divisions and could be transgenerationally extended. 

Another mode of modifying chromatin is through the substitution of canonical histone of nucleosomes for “histone variants”, the non-allelic paralogs of canonical histones. These changes modify the properties of nucleosomes altering chromatin. 

During spermatogenesis, histones are replaced by protamines that allow a higher compaction of chromatin in the nucleus of sperm head and facilitate the erasure of paternal epigenetic states. However, not all histones are replaced and some regions conserving histone scaffold can preserve their epigenetic marks and be potentially transmitted to their descendants via paternal transmission [46]. Additionally, male germ cell differentiation from PGCs requires established histone marks [47] followed by DNA methylation combined with changes in chromatin configuration and erasure of some histone post-translational modifications [48]. Chromatin structure and hence epigenetic changes could alter the regulation of specific gene expression in this key developmental window for gamete differentiation.

### 2.3. Non-Coding RNAs and Intermingled Epigenetic Mechanisms Involved in Transgenerational Transmission

The mechanisms of epigenetic modifications are not mutually exclusive but could be convergent or interactive. For example, very recently, Steward *et al.* [49] demonstrated that, in the mouse growing oocyte, the DNA methylation process at CpG islands is dependent on the remodeling of the histone marks, showing that H3K3 demethylation is necessary to proper DNA methylation. Consequently, environmental elements affecting histone marks could in turn induce some modification of the DNA methylation pattern. 

An increasing number of studies in germ cells reported the participation of ncRNAs in epigenetic inheritance of acquired alterations mediated by environmental conditions [50]. However, the mechanisms involved in the process are not yet fully established, but interaction of ncRNAs with classical epigenetic mechanisms including DNA methylation [51] and chromatin modifications by changing the histone patterns [52] have been suggested.

Some examples of transgeneracional transmissions of epigenetic changes with participation of ncRNAs in different pathways have been reported in animal models [52]. In *C. elegans*, Ashe *et al.* [53] demonstrated transgenerational epigenetic inheritance mechanisms, initially induced by piRNAs, with the participation of sncRNAs and chromatin pathways that can elicit a long-term epigenetic memory for more than 24 generations in germ cells. Additionally, piRNAs can also participate in epigenetic control of somatic cells regulating gene expression by interaction with chromatin structure, as demonstrated in *Aplysia* [54].

Environmental stressors, many of which are carcinogens or suspected carcinogens, are able to cause alterations in methylation and expression of transposable elements (TEs) initiating retrotransposition events. On the other hand, many methylation-regulated retrotransposon can escape from the global reprogramming of methylation and hence contribute to the inherited adverse phenotypes caused by reprotoxicants and environmental pollutants. Evidence summarized in this review suggests that TEs are the sensitive endpoints for detection of effects caused by such environmental stressors [55]. In this sense, piRNAs are highly ligated to the defense TEs in germ cells and zygotes [56]. TEs are constitutively associated with genome repetitive elements. Long-terminal-repeat-containing elements (LTRs), long interspersed nuclear elements (LINEs) and short interspersed nuclear elements (SINEs) are present in a wide variety of DNA methylation patterns in germ cells and preimplantation embryos. While LTRs display both hypermethylated and demethylated elements, LINEs are hypermethylated with elements escaping methylation and demethylation during preimplantation and SINEs are intermediate methylated in sperm followed by near complete hypomethylation over preimplantation [35]. In summary, these repeat elements display dynamic methylation processes. Consequently, combination of changes of methylation in repetitive elements for TEs and piRNAs-associated in gametogenesis and early embryogenesis [57,58], along with environmental changes methylation-related, could alter this complex equilibrium, modifying their epigenetic pattern. 

The acquisition of CpG islands and neo-methylable DNA regions during evolution and allele-specific parental genomic imprinting can be associated with genomic insertion of the CpG–rich transposable elements into the genome [59]. As insertion of transposable elements in the germ line is controlled by expression and activity of piRNAs, the regulation or alteration of piRNAs activity induced by environmental compounds during gametogenesis or during germ-cell fate can end in modifications of epigenetic landmarks by new CpG islands and potential new parental-specific imprinting.

Long non-protein coding RNAs (lncRNAs) are increasingly detected as relevant actors in development by new roles as mRNA regulators [60,61,62]. In mammalian cells, lncRNA are expressed from different genomic regions with different variant names and probably with diverse functions: from intergenic regions (lincRNAs), from gene introns (long-intronic ncRNAs), from the gene promoter regions (promoter-associated lncRNAs), from the opposite strand of mRNAs (antisense lncRNAs) or from pseudogenes [63].

The high inter-individual expression variability of lncRNAs detected in the same cell type from humans [64] suggests not only different epigenetic patterns in different tissues [65,66], but also potential changes environmentally induced, as was detected in sncRNAs. In this sense, it is open to question, for example, whether the biogenesis and functional structure of some sncRNAs are related to lncRNAs. Interestingly, 17.5% of human miRNA clusters were located in the genome in lncRNA regions that were defined as lnc-pri-miRNAs [67]. In the case of piRNAs, protein implicated in their biogenesis pathway have not yet well characterized, but there is evidence that many piRNA molecules were generated from genomic clusters (uni- and bi-directional) located at defined loci ranging from 1–100 kb in size [68,69,70]. Like lncRNA and miRNA clusters, piRNA clusters were transcribed by RNA polymerase II [71]. As mentioned above, a key function assigned to piRNA in germline was the repression of transposon expression. Interestingly, transposable elements are present in lncRNA sequences and play important roles in the lineage-specific diversification of lncRNA repertoires [72]. In mouse pre-gametic cells, the piRNA pathway controls the expression of both mRNA and lncRNA by the TEs present in both lncRNA and mRNA 3’UTR sequences [73]. Deep analysis of piRNAs in human adult testes showed that lncRNA could be a source of piRNAs [74], as we also detected in mouse gametes and zygotes. 

lncRNAs and piRNAs can be involved in mechanisms of histone modification. lncRNAs interact chromatin by recruitment of modifying enzymes to specific genomic loci able to change the chromatin state. An example of the relationship between lncRNAs and piRNAs in the landscape of epigenetic modifications of chromatin state and histone codes is the production of piRNAs by the lncRNA GAS5 that induce up-regulation of the TRAIL protein via H3K4/H3K27 methylation/demethylation [75]. The lncRNA HOTAIR (HOX Antisense Intergenic RNA), highly expressed in testes, is repressed by androgen, inhibits androgen receptor (AR) degradation and increases AR chromatin targeting [76]. HOTAIR is associated with the mammalian polycomb repressive complex 2 (PCR2) and interacting with chromatin and silencing *HOXD* gene, involved in metastasis suppression through H3K27 methylation and H3K4 demethylation locus [77]. The androgen or antiandrogen effect of many EDs is widely documented, and, consequently, a combined action of such EDs and this lncRNA could produce a synergistic action. Interestingly, both well known EDs—bisphenol-A and diethylstilbestrol—induce epigenetic alteration in HOTAIR promoters [78]. Additionally, some lncRNAs can interact with miRNAs acting as target of miRNAs [79] or as “molecular sponges” of miRNAs [80]. It was also reported that other EDs, such as the phytoestrogen genistein, inhibited *HOTAIR* by upregulation of *miR-34* which targeted HOTAIR results in tumor-suppressive roles [81]. About 40% of lncRNAs interact with chromatin by chromatin-modifying complex [82].

## 3. Are ncRNAs Sufficient for Transgenerational Epigenetic Inheritance of Environmental Induced Characters?

In most of the reported cases of parental transmission of phenotypes induced by environmental reprotoxicants, epigenetic changes of DNA methylation and chromatin modifications of germ cells from exposed parents have been pointed out as the main mechanisms involved. Reprotoxicants assessed include some well-known endocrine disruptor compounds, as is the widely used antiandrogen fungicide: vinclozolin [83,84,85]. However, increasing studies are showing an active participation of different ncRNAs in epigenetic inheritance unrelated to DNA methylation or histone modifications. In fact, in male mice of F1 generation exposed *in utero* to vinclozolin until the day 13.5 *post coitum*, we detected reduction in the number of embryonic PGCs and an increased rate of apoptotic cells along with a decrease of fertility rate in adult males from F1 to successive generations until F3. Along with these transgenerationally transmitted phenotypic disorders, specific microRNAs expressed in PGCs were detected as significantly overexpressed: *miR-23b* and *miR-21*. This increase of these specific miRNA induced disequilibrium in the *Lin28*/*let-7*/*Blimp1* pathway, a crucial regulator of PGC differentiation, that was deregulated in three successive generations of males not exposed to the compound. However, interestingly, all these epigenetic inherited characters, detected in PGCs at the levels of gene expression, protein, cellular and reproductive traits were not accompanied by changes in DNA methylation in PGCs or in mature sperm [86]. These data could be considered as representative of the direct participation of a type of sncRNA, miRNA in this case, in the epigenetic transmission of environmental induced effects of reprotoxicants, through successive generations via paternal transmission [24].

As suggested above, piRNAs are emerging as the most versatile and malleable sncRNAs. Very recently, tRNA-derived fragments (called tsRNAs in the reports) have been identified as sperm-borne molecules involved in the transmission of acquired metabolic disorders without direct participation of DNA methylation changes [87]. Curiously, similar sncRNAs tRNAs-derived preferentially from the 5′ halves of mature tRNAs were also detected in unicellular organisms such trypanosomids, after nutritional stress [88]. However, the authors of this paper and others have identified these types of so-called tsRNAs as piRNAs potentially generated from the processing of different tRNAs in germ cells [57,58,89] and human cancer cells [90,91]. Thus, since these peculiar tRNA-derived or tRNA fragments (tRFs) could be functional piRNAs transmitting environmentally induced phenotypes, it is possible to speculate that both tRNA and piRNA pathways involved in their biogenesis could participate in epigenetic mechanisms that deserve further investigations.

**Figure 1:**
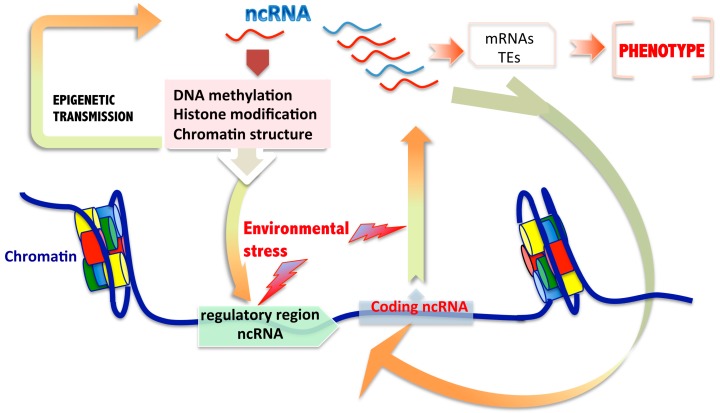
Schematic model of proposed mechanisms of epigenetic transmission of changes induced in ncRNAs by effects of environmental reprotoxicants. ncRNAs could contribute to inherit changes to successive generations through germ cells. This suggested model could involve different putative actors well-known as participants in epigenetic changes environmentally-mediated, including the effects of reprotoxicants.

How can an epigenetic transgenerational inheritance of changes in ncRNA expression be explained when the cause of the disturbance is in themselves? We hypothesize a model in which, independently of the type of ncRNA, any alteration of their expression or their functional targeting could be initiated in the genome regulatory region of ncRNAs affecting the level of expression and/or by post-transcriptional modifications during their biogenesis processes. These individual or combined effects could induce alteration chromatin structure, changes in DNA methylation or histone patterns in themselves gene regulatory regions, or in others. Such modifications could be epigenetically transmitted to successive generations (Figure 1). Additionally, the changes in the targets of ncRNAs (Vg: mRNAs or transposable elements) could also in turn impact the regulation of ncRNAs themselves.

## 4. RNA Epigenetics: A New Player Emerging on the Scene

Additional mechanisms of post-transcriptional regulation of gene expression have recently been reported by modifications of RNA molecules. RNA sequencing by NGS is providing the identification of multiple types of such epigenetic modifications in diverse classes of RNAs: mRNA, tRNAs, sncRNAs and lncRNAs. These include methylation of adenosine residues at the N6 position (m^6^A) by methylation protein METTL3, of which a lack can affect global gene expression [92]. Environmental stress conditions are able to induce m^6^A methylation of mRNAs, controlling stress protein expression such as Hsp70 [93]. RNA epigenetic marks as key elements in the developmental program have been recently verified. Early differentiation in mice is affected by changes in the pattern m6A marks mediated by METTL3, resulting in embryonic lethality [94,95]. This point is important to consider along with other epigenetic marks in relation to the most sensitive developmental windows during early embryogenesis periods, as is pointed out here for other potential epigenetic effects caused by environmental stress. The potential effects of stressors in RNA epigenetic modifications with evident phenotypes have bidirectional nature. RNA marks as methylation can alter the biogenesis of sncRNAs and change the specification of potential functions such as mRNA targets of defined miRNAs, or the modified mRNAs that could be regulated post-transcriptionally by other miRNAs. In turn, m6A can be regulated by miRNAs and affect the stem cell fate [96]. However, adenosine methylation is just a well-documented example of potential epigenetic modifications of RNAs. Alterations of other RNA editions such as cytidine to uridine in specific RNAs can cause testicular germ cell tumors under potential adverse environmental influences [97].

Also affecting adenosine, the RNA editing A-to-I, is the deamination of adenosine by the ADAR family of proteins to a modified base such as inosine (I), which is recognized as guanosine by the cells [98,99]. This epigenetic mark can be generated in the pre-mRNA but mainly in miRNA precursors during their biogenesis process due to the high affinity of ADAR proteins to double stranded RNA. The expression of *Adar* is mainly in testes and the brain [100,101,102]. The A-to-I editing is a reprogrammable process, as we have reported in mouse during preimplantation embryogenesis [103]. Consequently, environmental changes are able to modify the regular pattern of RNA edition. These modifications can clearly change the potential targets of miRNA and hence the corresponding gene expression pattern [104]. As the RNA editing, ADAR-mediated, is high in testes, environmental exposure to reprotoxicants during gonadal development and germ cell differentiation could be involved in some of the TDS entities including testicular cancer or infertility. Epigenetic changes in RNA A-to-I editing in *Drosophila* as a consequence of environmental adaptations were also discovered [105]. The potential epigenetic effects of alterations in the A-to-I RNA editing has also been recently reported in the recoding of mRNAs of 22 genes involved in neurodegenerative diseases such as Alzheimer's disease [106].

Interestingly, a new potential RNA modifications with putative roles in epigenetic changes through sncRNAs could be mediated by epigenomic changes in post-transcriptional modifications affecting tRNAs and piRNA-tRNA-derived, such as N^1^-methyladenosine (m^1^A) [107], 5-methylcytidine (m^5^C) or N^2^-methylguanosine (m^2^G), as were recently reported in tsRNAs (presumptively piRNAs) detected in the sperm of mice exposed to high-fat diets that were injected into zygotes that transmit metabolic disorders to the F1 offspring [87].

Most of the inherited epimutations detected both intergenerationally and transgenerationally were caused by acute exposure during a defined window in the development. However, a great number of environmental exposures to reprotoxicants (as the increasing rate of pollution by EDs) could be considered chronic exposure. It is logical to speculate on the possibility that some of the primary epigenetic effects can be evolving to “tertiary epimutation” [108] or genetic mutations as a cause of prolonged exposure promoting genome instability [109], even in successive generations. 

A combination of *in vivo* and *in vitro* approaches could improve in the future our knowledge of the mechanisms involved in the role of ncRNAs in the transgenerational transmission of the diverse epigenetic acquired traits in both sexes. Additional to the direct effects of environmental toxicants upon germ cell differentiation and function, indirect effects by ncRNA-mediated should also be considered. The possibility of circulating molecules of ncRNA—for example, in exosome-like vesicles—should be taken into account to be investigated in the near future as potential cross-talk mechanisms altering germ cells after functional deregulation of ncRNA in other organs, such as the liver. These potential actions of “circulating ncRNAs” could be evaluated in the near future by implementation of *in vitro* methods of germ cell differentiation.

## 5. Conclusions

ncRNAs could represent key regulatory elements in the epigenetic transgenerational transmission of the effects of reprotoxicants on germ cell differentiation and development and consequently in the decrease of fertility in mammals as consequence of the action environmental pollutants in early development.
